# Protocolized abuse screening to decrease provider bias and increase capture of potential events

**DOI:** 10.1186/s40621-024-00495-4

**Published:** 2024-03-28

**Authors:** Ashley Chan, Mary D. Feller, Kaylin Dawson, Kirsten Morrissey, Ashar Ata, Mary J. Edwards

**Affiliations:** 1 School of Medicine, Albany Medical College, Albany, NY USA; 2 Department of Emergency Medicine, Albany Medical College, Albany, NY USA; 3 Department of Surgery, Albany Medical Center, 2d Floor, 50 New Scotland Avenue, Albany, NY 12208 USA

**Keywords:** Child abuse, Screen, Race, Poverty, Insurance

## Abstract

**Background:**

Early identification of child abuse is critical to prevent death and disability. Studies suggest implicit bias of providers may lead to overrepresentation of minority and impoverished children in child abuse reporting. At our institution, universal screening for sexual and physical abuse for all children under 18 years of age was implemented in 2016. A rigorous, objective evaluation protocol focusing on the mechanism of injury and exam findings to improve recognition and eliminate bias was implemented in 2019.

**Findings:**

Demographics and clinical characteristics of patients less than 18 years of age were abstracted by chart review (2014–2015) and from a forensic database (2016–2022). International Classification of Diseases codes 995.5 (version 9) and T76.12XA (version 10) were used to identify patients before the establishment of forensic database. Relative frequency and patient characteristics of the three time periods (pre universal screening: 2014–2015, post universal screening: 2016–2019, post protocol implementation: 2020–2022) were compared using Chi-square tests and modified Poisson regression. Universal screening significantly increased the number of cases identified. The demographic profile of potential victims by race significantly changed over the reporting periods with an increased number of white children identified, consistent with state demographics. The proportion of publicly insured patients trended down with universal screening and protocol implementation, despite a significant increase in the number of children publicly insured in the state during this time.

**Conclusion:**

These single institutional results lend support to objective, evidence-based protocols to help eliminate bias surrounding race and poverty.

**Supplementary Information:**

The online version contains supplementary material available at 10.1186/s40621-024-00495-4.

## Introduction

Every year in the United States millions of reports of suspected child abuse are referred to state and local Child Protective Services. In 2020, 17% of these reports nationwide were substantiated (U.S. Department of Health & Human Services, Administration for Children & Families 2022). This devastating problem has lifelong physical and mental ramifications for children and their families (Flaherty et al. 2013; Hoft et al. 2017). The Centers for Disease Control estimates that in 2021 one in seven children in the United States experienced child abuse or neglect, and that in 2018 the total lifetime economic burden surrounding abuse and neglect was 592 billion dollars (https://www.cdc.gov/violenceprevention/childabuseandneglect/fastfact.html). Early identification of abuse is critical for a child’s future health and safety. A significant body of evidence surrounding the cumulative effect of adverse childhood experiences demonstrates that early intervention and prevention will have an exponentially positive effect on future health of families and conservation of healthcare resources (https://preventchildabuse.org/resources/adverse-childhood-experiences-robert-anda/).

When a child presents with injury, the decision to embark on an investigation of abuse historically rests on provider discretion with inherent risk of implicit bias. Implicit bias is unconscious or conscious attitudes we all hold against others due to past experiences (Shah and Bohlen 2024).

The impact of implicit bias in abuse evaluations has been demonstrated in multiple studies demonstrating overrepresentation of minority (particularly Black and Hispanic) children and children with public health insurance in reports to Child Protective services (Higginbotham et al. 2014; Johnson-Motoyama et al. 2022; Joseph et al. 2022). Although poverty is a known risk factor for physical abuse, a reporter’s bias not only can lead to unnecessary burdens on minority families, but a potential for missed cases in more affluent patients due to failed recognition (Rebbe et al. 2022).

In response to a requirement for “a mechanism for assessment of abuse” by the American College of Surgeons for our pediatric trauma center verification (Resources for Optimal Care of the Injured Patient 2014), our institution introduced universal screening for abuse for all children in 2016. Screening is based on 7 questions obtained at presentation. As six of the questions are prone to subjectivity, in 2019 an evidence-based protocol was developed to objectively introduce evaluations based positive screens and “red flag” injuries. Activation of this protocol prompts a forensic nursing evaluation and a battery of imaging and laboratory tests based on age and clinical exam (see Additional file 1).

We hypothesized that implementation of universal screening would increase the number of cases identified and referred to Child Protective Services, and the implementation of an objective protocol would change the overall proportion of minority and publicly insured families to be more representative of the region’s overall demographics.

## Methods

**Fig. 1:**
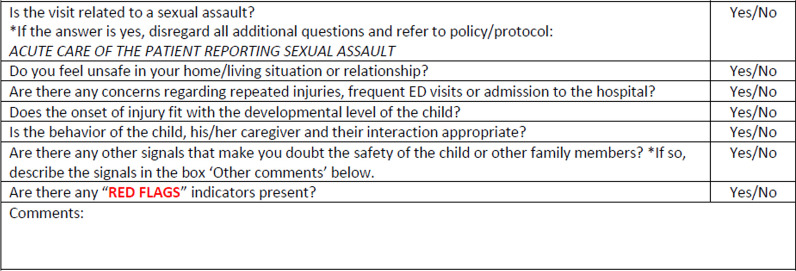
Universal screening questions

**Fig. 2:**
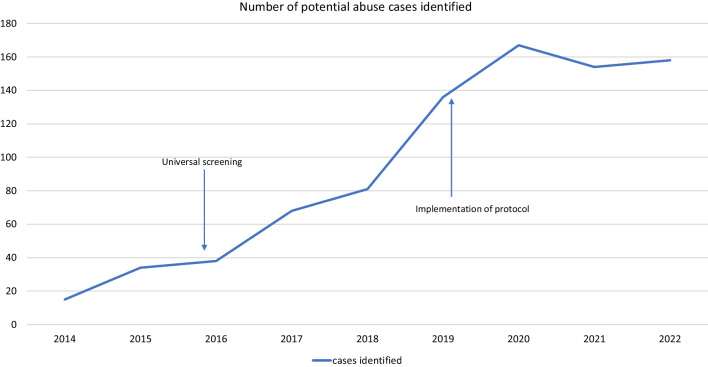
Number of potential victims identified by year

The screening process established in 2016 stipulates that all patients under the age of 18 years are screened for physical and sexual abuse regardless of point of entry to the hospital. This is a series of 7 questions that the nurse screening the patient either asks the patient or assesses his or herself (Figs. 1 and 2). In the event of a positive screen, the nursing staff will alert the unit’s social worker and attending physician for a “maltreatment huddle.” At this point the decision will be made to proceed along the maltreatment clinical pathway. This pathway includes an evaluation by a forensic nurse examiner, a report to Child Protective Services by social work, and a series of lab and imaging studies based on the age of the child and physical exam findings. A maltreatment huddle does not necessarily have to be done initially, but at any time during the patient’s care if concerns arise.

In 2019, an evaluation protocol was used to create a standardized order set. This is consistent with the best practices as outlined by the 2019 American College of Surgeon’s Best Practice Guidelines for Trauma Center Recognition of Child Abuse, Elder Abuse, and Intimate Partner Violence (American College of Surgeons 2019). Order set is available for review in the Additional file 1.

The study period was divided in three categories based on how potential abuse patients were screened: (a) pre-universal screening, mainly based on provider discretion (2014–2015); (b) post universal screening (2016–2019); (c) universal screening with the above protocol (2020–2022). For the pre universal screening period, patients were identified using International Classification of Diseases codes representing suspected child physical abuse—995.5 (version 9) and T76.12XA (version 10). Data on patient demographics (including age, race, gender, insurance) and clinical characteristics (including primary diagnosis, discharge disposition) was collected by review of electronic medical charts. For the latter two time periods the prospective forensic database established after 2016 was used to identify the study patients. The forensic database pulls similar demographic data from the health records but does not rely on ICD codes to identify potential victims. All patients seen by the forensic nurse examiner team are entered into this prospective database.

Relative frequency and patient characteristics of the three time periods were described using frequencies and percentages and compared using Chi-square tests. Modified Poisson regression was used to estimate Incident Risk Ratios quantifying the effect of screening methods on the demographic composition of the patients in the database. Demographics and insurance status were then compared to the overall estimated census data for New York state in 2022 and the Department of Health records for public insurance (U.S. Census Bureau 2022).

## Results

The number of cases evaluated per year for potential abuse increased significantly over time after the implementation of universal screening (Fig. 1). Protocol implementation did not significantly increase or decrease the number of cases identified in consecutive years. By means of comparison, the Emergency Department (ED) did demonstrate some overall growth during this time, but not enough to account for the changes in cases identified during time periods 1 and 2. Average annual pediatric ED visits prior to protocol implementation (time 1) were 17, 101, after protocol implementation during period 2 they were 19, 547 (a 14% increase), and for time period three were 21, 273 (a 9% increase).

The racial distribution of children identified as potential victims of abuse changed significantly between the three periods (Table 1). The proportion of white children trended up after universal screening. As compared to pre-universal screening, evaluated children were 1.71 (95% CI 1.15, 2.54) times more likely to be white in the universal screening period and 1.47(0.99, 2.19) times more likely to be white in the post protocol implementation period. The proportion of Hispanic and Asian children trended down over time. The proportion of patients with an “unknown or other” race fell significantly from 24.5 to 10.2% and 16.3% in the two successive periods after universal screening and with implementation of a dedicated forensic database for quality improvement. The racial breakdown of children in this cohort trended closer to the overall racial profile of residents in the state during this period (54% white, 17% Black, 20% Hispanic, 10% Asian) (Resources for Optimal Care of the Injured Patient 2014).

**Table 1:** Racial breakdown of potential victims by study time period

Period	White	Black	Hispanic	Asian	Other/unknown
2014–2015 (n = 49)	17(34.7%)	12 (24.5%)	5 (10.2%)	3 (6.1%)	12 (24.5%)
2016–2019 (after universal screening, n = 323)	192 (59.4%)	77 (23.8%)	16 (5%)	5 (1.5%)	33 (10.2%)
2020–2022 (after protocol implementation, n = 479)	245 (51.1%)	124 (25.9%)	19 (4%)	13 (2.7%)	78 (16.3%)

*P* = 0.004

The proportion of children publicly insured trended down over the three periods, but this did not reach statistical significance (Table 2). However, this was despite a 33% increase in residents covered under the state’s public health insurance program during this time (Healthinsurance.org).

**Table 2:** Breakdown of potential victims by insurance carrier over study time period

Period	Public insurance	Other
2014–2015 (n = 49)	41 (83.7%)	8 (16.3%)
2016–2019 (after universal screening, n = 323)	264(81.7%)	59 (18.3%)
2020–2022(after protocol implementation, n = 479)	372 (77.7%)	107 (22.3%)

*P* = 0.285

## Discussion

This study demonstrates the impact in one institution of universal screening and an objective, injury-based reporting protocol on the proportion of minority and socioeconomically disadvantaged children identified as potential victims of abuse. Universal screening and protocol implementation had a significant impact in increasing the number of potential victims identified which outpaced overall changes in ED workload during this time. These changes also shifted the demographics of potential victims to be more consistent with the overall profile of the state’s population.

Provider implicit bias is inherently unconscious, automatic, and universal. The associations between provider implicit bias and healthcare outcomes are well established (Maina et al. 2018). It follows that a process which depends heavily on subjective suspicions of a provider would be subject to significant racial inequities in identifying of child abuse. Previous studies have demonstrated the worrisome impact of provider bias in the evaluation and reporting of child abuse (Hymel et al. 2018). Specifically, a missed abuse diagnosis is more likely in a white child and skeletal surveys to identify occult fractures are more likely to be completed in underrepresented minority children (Jenny et al. 1999; Lane et al. 2002). In this study, we found that universal screening increased the number of white and privately insured children identified as potential victims of abuse. These align with the results of a like study done in Austin, Texas where universal screening of children under age one year with fractures resulted in more privately insured patients being screened (Higginbotham et al. 2014). Our objective pathway results are similar to a Connecticut study where certain injuries in infants immediately prompted consultation with Child Protective Services and Social Work. This intervention resulted in a relative decrease in the number of publicly insured children being evaluated for abuse (Powers et al. 2019).

Although numerous studies clearly demonstrate that minority and publicly insured children are more likely to be investigated for abuse, ours is one of the few that demonstrates the impact of universal screening and an objective protocol (Joseph et al. 2022). Unlike the Texas study, in our cohort, all children presenting the children’s hospital were screened. Not surprisingly, the total number of identified cases increased with universal screening, and the racial proportions significantly changed, with more white children identified as potential victims.

In terms of feasibility and generalizability of this intervention, universal screening did not significantly impact hospital workflow. This became a standard part of nursing intake screening at point of entry to the inpatient setting or emergency department. However, as the number of potential cases identified increased, more resources were required to complete the evaluations. These included radiology resources for skeletal surveys and CT scans, forensic nursing resources for documenting physical examinations with photos, ophthalmology exams, and social work resources to communicate with CPS. One of the benefits of the order set and evidence-based protocol was to decrease the unnecessary imaging studies and exams to what was truly needed based on the patient’s age and presentation.

This study has several limitations. Prior to 2016 screening was not universal and data capture of suspected abuse in the electronic health record was reliant on ICD 9 and 10 codes. This likely resulted in missed cases as communications with child protective services could not be searched for accurately in existing financial or trauma databases. After 2016 a separate forensic nursing database was established and likely significantly improved data capture. This likely explains the significant decrease in the “unknown” racial category over time. The sample population is representative of the region served by Albany Medical Center (rural and suburban) and may not be generalizable to the entire state, as much of the New York metropolitan area is not represented. The low sample size likely impacted statistical analysis and generalizability, particularly prior to 2016. The use of public health insurance as a surrogate for low socioeconomic status has been previously validated (Snyder and Chang 2020), but has its own limitations as well. Finally, given privacy laws we were unable to definitively determine retrospectively which patients that screened positive had confirmed cases of abuse. Beginning in 2023 we prospectively began recording changes in caregiver status at the time of discharge as a surrogate for a confirmed positive case of abuse.

Despite the limitations, this study is important as it lends further evidence to the utility of a dedicated universal screening protocol to identify children who are victims of abuse. The results stress the importance of further studies to study the impact of universal screening protocols in a variety of clinical settings.

## Conclusions

After an evidence based, universal protocol for child abuse screening was implemented the percentage of white children identified as potential victims increased significantly and the number privately insured trended higher. These results lend support to objective, evidence-based protocols to help eliminate bias surrounding race and poverty. This study supports a growing body of knowledge that universal screening and objective evaluations focused on injuries, mechanism and patient presentation can help mitigate the healthcare disparities seen in communities resulting from implicit bias and structural racism.

### Supplementary Information

**Additional file 1.** Abuse Screening and Evaluation Protocol.

## Data Availability

The datasets used and analyzed during the current study are available from the corresponding author on reasonable request.

## Abbreviation

ICD: International classification of diseases

## References

[CR1] American College of Surgeons Trauma Quality Improvement Program Best Practice Guidelines for Trauma Center Recognition of Child Abuse, Elder Abuse and Intimate Partner Violence. Published 11/2019. Available at: https://www.facs.org/media/o0wdimys/abuse_guidelines.pdf

[CR2] Flaherty EG, Thompson R, Dubowitz H, Harvey EM, English DJ, Proctor LJ, Runyan DK (2013). Adverse childhood experiences and child health in early adolescence. JAMA Pediatr.

[CR3] Healthinsurance.org. https://www.healthinsurance.org/medicaid/new-york/. Accessed 12/7/2023

[CR4] Higginbotham N, Lawson KA, Gettig K, Roth J, Hopper E, Higginbotham E, George TM, Maxson T, Edwards G, Garcia NM (2014). Utility of a child abuse screening guideline in an urban pediatric emergency department. J Trauma Acute Care Surg.

[CR5] Hoft M, Haddad L (2017). Screening children for abuse and neglect: a review of the literature. J Forensic Nurs.

[CR6] https://www.cdc.gov/violenceprevention/childabuseandneglect/fastfact.html Accessed 1/23/2023.

[CR7] https://preventchildabuse.org/resources/adverse-childhood-experiences-robert-anda/ Accessed 1/23/2023

[CR8] Hymel KP, Laskey AL, Crowell KR, Wang M, Armijo-Garcia V, Frazier TN, Tieves KS, Foster R, Weeks K, Dias MS, Halstead ES (2018). Racial and ethnic disparities and bias in the evaluation and reporting of abusive head trauma. J Pediatr.

[CR9] Jenny C, Hymel KP, Ritzen A, Reinert SE, Hay TC (1999). Analysis of missed cases of abusive head trauma. JAMA.

[CR10] Johnson-Motoyama M, Ginther D, Oslund P (2022). Association between state Supplemental nutrition assistance program policies, child protective services involvement and foster care in the US, 2004–2016. JAMA Netw Open.

[CR11] Joseph B, Sakran JV, Obaid O, Hosseinpour H, Ditillo M, Anand T, Zakrison TL (2022). Nationwide management of trauma in child abuse: exploring the racial, ethnic, and socioeconomic disparities. Ann Surg.

[CR12] Lane WG, Rubin DM, Monteith R, Christian CW (2002). Racial differences in the evaluation of pediatric fractures for physical abuse. JAMA.

[CR13] Maina IW, Belton TD, Ginzberg S, Singh A, Johnson TJ (2018). A decade of studying implicit racial/ethnic bias in healthcare providers using the implicit association test. Soc Sci Med.

[CR14] Powers E, Tiyyagura G, Asnes AG, Leventhal JM, Moles R, Christison-Lagay E, Groisberg S, Auerbach M (2019). Early Involvement of the Child Protection Team in the Care of Injured Infants in a Pediatric Emergency Department. J Emerg Med.

[CR15] Rebbe R, Sattler KM, Mienko JA. The association of race, ethnicity, and poverty with child maltreatment reporting. Pediatrics. 2022;150(2). 10.1542/peds.2021-053346 PMC11590070 35843980

[CR16] Resources for Optimal Care of the Injured Patient (2014). American College of Surgeons, Chicago, Il, 2014. p 68

[CR17] Shah HS, Bohlen J. Implicit Bias. [Updated 2023 Mar 4]. In: StatPearls [Internet]. Treasure Island (FL): StatPearls Publishing; 2024 Jan-. Available from: https://www.ncbi.nlm.nih.gov/books/NBK589697/36944001

[CR18] Snyder RA, Chang GJ (2020). Insurance status as a surrogate for social determinants of health in cancer clinical trials. JAMA Netw Open.

[CR19] U.S. Census Bureau 2022 Population Estimates, New York State https://www.census.gov/quickfacts/fact/table/NY/PST045222. Accessed 12/7/2023

[CR20] U.S. Department of Health & Human Services, Administration for Children & Families. Child Maltreatment 2020. 2022. https://www.acf.hhs.gov/cb/report/child-maltreatment-2020. Accessed Dec 12, 2023.

