# Supplementary material for: Protocolized abuse screening to decrease provider bias and increase capture of potential events
Source: Inj Epidemiol. 2024 Mar 28;11:11. doi: 10.1186/s40621-024-00495-4 (PMC10976801; doi:10.1186/s40621-024-00495-4)
Supplement: Supplementary file 1 — Additional file 1. Abuse Screening and Evaluation Protocol. [file 40621_2024_495_MOESM1_ESM.pdf]

ALBANY MED

Pediatric Maltreatment Protocol

Overview

Albany Medical Center Hospital is committed to maintaining an environment that ensures all patients are appropriately screened for safety. Accordingly, the Hospital has established a protocol for the care and treatment of the pediatric patient (<18 years) presenting with injury or condition suspicious for maltreatment. This protocol is based on current guidelines and is consistent with all applicable laws and regulations pertaining to mandated reporting of suspected child abuse/neglect.

Objectives

- Standardize the clinical practice in cases of suspected maltreatment that present to Albany Medical Center
- Provide clear, evidence- based guidelines for ordering laboratory and radiographic testing when maltreatment is suspected
- Decrease the ordering of unnecessary imaging studies
- Reduce bias in the evaluation of suspected maltreatment
- Meet the patient's medical, emotional, and legal needs

Definitions

Mandated Reporter	Mandated reporters are required to report suspected child abuse or maltreatment when, in their professional capacity, they are presented with reasonable cause to suspect child abuse or maltreatment. In the hospital environment, this includes all clinical staff engaged in the admission, examination, care, or treatment of the patient.
Reasonable Cause	Reasonable cause to suspect means the belief that based on personal observation or relay of information combined with training and experience, harm or imminent danger of harm to the child has or could result.
Physical Abuse	Any non-accidental injury to a child.
Neglect	The failure of a parent or other person with responsibility for the child to provide needed food, clothing, shelter, medical care, or supervision to the degree that the child's health, safety, and well-being are threatened with harm.
Sexual abuse	Involvement of a child in sexual activity that he or she does not fully comprehend, is unable to give informed consent to, or for which the child is not developmentally prepared and cannot give consent, or that violates the laws or social taboos of society.
Maltreatment Huddle	Collaboration amongst health care providers when a pediatric physical maltreatment case has been identified. Huddles can occur at any point in the health care stay but should be done on a daily basis.

Process & Procedure

All pediatric patients presenting to AMCH will be screened for maltreatment regardless of entry point to the hospital.

1. In Soarian, complete the ***Maltreatment Screening Tool for Pediatric Patients (<18 years)***:

Is the visit related to a sexual assault?<br>*If the answer is yes, disregard all additional questions and refer to policy/protocol:<br>*ACUTE CARE OF THE PATIENT REPORTING SEXUAL ASSAULT*	Yes/No
Do you feel unsafe in your home/living situation or relationship?	Yes/No
Are there any concerns regarding repeated injuries, frequent ED visits or admission to the hospital?	Yes/No
Does the onset of injury fit with the developmental level of the child?	Yes/No
Is the behavior of the child, his/her caregiver and their interaction appropriate?	Yes/No
Are there any other signals that make you doubt the safety of the child or other family members? *If so, describe the signals in the box 'Other comments' below.	Yes/No
Are there any "**RED FLAGS**" indicators present?	Yes/No
Comments:	

2. Identify any "**RED FLAGS**": (*the below list is not all inclusive*)

a. Evaluating History of Present Injury
- No history or inconsistent history
- Changing history
- Unwitnessed injury
- Delay in seeking care
- Domestic violence in home

B.

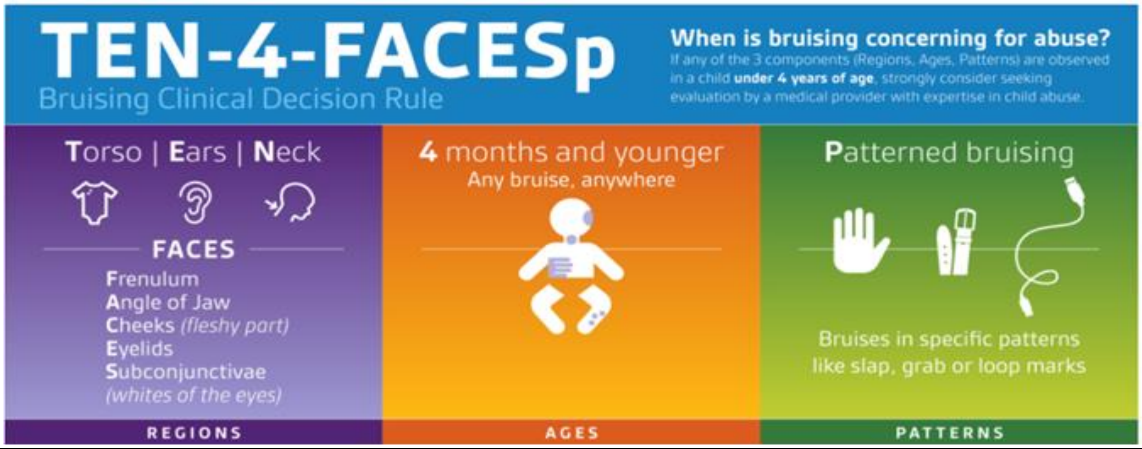

c. Radiographic Findings
- Metaphyseal fractures (corner)
- Rib fractures (especially posterior)
- Any fracture in a non-ambulatory infant
- An undiagnosed healing fracture(s)
- Any intracranial hemorrhage in a child <3 years of age without a consistent history of significant trauma, in the absence of a skull fracture, or no history

d. Consideration for Radiographic Finding Drop Off Criteria
- Distal radial or ulnar buckle fracture in a cruising child > 9 month with a history of a consistent fall
- Linear, unilateral, non-depressed skull fracture in a child > 6 month with a history of a significant fall and no additional clinical concerns
- Unilateral Clavicle fracture likely attributed to birth (acute fracture in infants < 22 days or healing fracture in infants < 30 days)

3. If a **POSITIVE SCREENING** is identified:
    a. In collaboration with the health care team (Attending Physician, Resident Physician, Midlevel Provider, Social Work), the RN will initiate the Pathway for Suspected Pediatric Maltreatment (see Attachment A). A maltreatment huddle must occur amongst all providers caring for the patient. This is documented in the nursing assessment "Maltreatment huddle". A maltreatment huddle can occur multiple times throughout the hospitalization and should be documented accordingly.
    b. The provider will complete the Suspected Pediatric Maltreatment Order Set (see Attachment B).
    c. The licensed social worker is responsible for notifying Child Protective Services (CPS), however any member of the health care team may report in the event social work is not immediately available.
    a. The primary RN will notify the Pediatric ED Charge RN (264-5437) to contact the forensic examiner on-call. If the patient is admitted directly to the floor, it is the primary RN's responsibility to call the Pediatric ED Charge RN to activate the forensic examiner. If the patient is on the floor, and a forensic/sexual assault exam is indicated, it is the primary RN's responsibility to call the pediatric ED Charge RN (264-5437).
    b. The role of the forensic examiner is to obtain the history, document injury and/or lack thereof, photograph injuries if present, and complete a physical assessment. In a physical abuse case, there may be an opportunity to collect forensic evidence. In such case the examiner will follow all appropriate processes. It is the role of the examiner to communicate with all members of the team when the exam is complete. The forensic examiner will initiate the Pediatric Maltreatment Checklist (Attachment C).

4. Communication with Parent/Guardian(s):
    a. In collaboration with the health care team (Attending Physician, Resident Physician, Midlevel Provider, Social Work, Nursing), the parent/legal guardian(s) may be informed if a referral to Child Protective Services (CPS) has been filed and/or law enforcement has been notified.

        Parent/Guardian(s) may be informed as follows:
        "Any time a child of this age presents to the hospital with (identify injury), we evaluate for additional injuries. Sometimes a child may have internal conditions such as fractures, head or abdominal injuries that we cannot see on the outside. Just like you, we want to make sure your child is okay, so it is important that we do this testing. These tests include (specify testing). We will also have our social worker come talk to you. This is a standard and universal part of our evaluation and we are happy to answer any questions or concerns along the way."
    b. Communication should be direct and objective. Inform parent/guardian(s) that inflicted trauma is part of the diagnostic consideration. Discuss with them the evaluation of the forensic examiner.
        i. For the patient that there is an indication for a sexual assault examination refer to the *Acute Care of the Sexual Assault Protocol:* https://intranet.amc.edu/display/NURSING/Acute+Care+of+the+Sexual+Assault+Protocol
    c. Maintain focus on the child and avoid appearing judgmental. Assure parents of the need for thorough evaluation.
    d. Seek assistance from Social Work prior to and during the conversation.
    e. In the rare circumstance where the health care team determines it is not in the patient's best interest to notify parent/guardian(s) of a CPS referral, communication among the team and during hand off to the unit (if applicable) is critical.

5. Notification to Child Protective Services (CPS):
    a. The licensed social worker will act as the mandated reporter for the health care team and notify CPS of any suspected child abuse. In addition, a CPS report can be made by <u>any</u> member of the health care team. Confirmation is not needed for a CPS investigation. The mandated CPS report hotline is: **1-800-635-1522.**
    b. The preferred medical provider to communicate with Child Protective Services is the attending physician. This can be coordinated with the assistance of Social Work.
    c. When speaking to Child Protective Services, a clear description of the injuries and reasons for medical concern of maltreatment should be discussed. Avoid using the terminology “patient cleared.” Using the term “patient medically ready for discharge” should be encouraged.
    d. When patients are transferred to AMC with suspicion of maltreatment, the social worker will notify CPS of the arrival of the patient and complete the online reporting regardless of whether the transferring facility has filed a CPS report. When CPS accompanies a child to AMC, a report from AMC should be generated to be the source of the report and relay all necessary information to them.
    e. Law enforcement will only be notified at the request of the patient and/or consent of the parent/guardian(s), with the following exceptions:
        i. gunshot wound, powder burns and other injuries caused by the discharge of a gun, and stabbing injuries which may result in death
        ii. CPS will notify law enforcement as per CPS policy
    f. The treating physician may notify CPS or appropriate law enforcement to take custody of a pediatric patient whether or not additional medical treatment is required if the physician has reasonable cause to believe that the circumstances or condition of the child are such that continuing in the care and custody of the patient/ parent/guardian(s) presents an imminent danger to the child's life or health.
6. Release of Information
    a. If AMCH is the source of the CPS report, the patient's medical records relevant to the subject encounter may be released to CPS.
    b. PHI may be released to law enforcement with a signed HIPPA authorization (signed by the patient/guardian(s), or court-ordered subpoena
    c. PHI may be disclosed to law enforcement without a signed HIPPA authorization in certain incidents including:
        i. Releasing of information to prevent or lessen a serious or imminent threat to the health or safety of an individual or public
        ii. Releasing of information when required by law to do so (such as reporting gunshot or stab wounds)
7. Disposition from Emergency Department
    a. If any suspicion of maltreatment/non-accidental trauma has been raised during the ED visit, all members of the health care team should participate in a face-to-face huddle.
    b. All physical maltreatment evaluations that require admission will be admitted to Trauma/Pediatric surgery service.
    c. Prior to disposition from the hospital, children who have undergone a child maltreatment evaluation MUST have final radiology interpretations in the medical record of all imaging studies obtained, and documentation of attending oversight on all consultations. For ophthalmology consultations must be by a faculty ophthalmologist.
    d. If the patient is discharged to home, communication with all members, and CPS must be completed prior to the discharge to ensure a safe discharge.
    e. It is recommended that outpatient follow-up be made upon discharge with a CHAMP-certified physician. In addition, follow up studies may be recommended by the CHAMP certified physician.

8. Disposition from Hospital Admission
    a. Prior to hospital discharge, all members of the health care team must be made aware of the disposition of the patient and in collaboration with CPS plan for discharge.
    b. Prior to disposition from the hospital, children who have undergone a child maltreatment evaluation MUST have final radiology interpretations in the medical record of all imaging studies obtained, and documentation of attending oversight on all consultations. For ophthalmology consultations must be by a faculty ophthalmologist.
    c. It is recommended that outpatient follow-up be made upon discharge with a CHAMP-certified physician. In addition, follow up studies may be recommended by the CHAMP certified physician.

ASSOCIATED REFERENCES

- N.Y. Soc. Serv. Law §§ 412, 413
- AMCH Policies:
    - *Acute Care of the Patient Reporting Sexual Assault*
    - *Assessment and Treatment of Victims of Abuse, Neglect or Exploitation*
    - *Reportable Diseases and Conditions*
- http://pediatrics.aappublications.org/content/141/1/e20171994?utm_source=highwire&utm_medium=email&utm_campaign=Pediatrics_etoc
- https://ocfs.ny.gov/main/cps/
- Henry MK, Bennett CE, Wood JN, "Evaluation of the abdomen in the setting of suspected child abuse." Pediatric Radiology 2021.
- Pierce MC, Kaczor K, Lorenz DJ, et al. Validation of a Clinical Decision Rule to Predict Abuse in Young Children Based on Bruising Characteristics. *JAMA Netw Open.* 2021;4(4):e215832. doi:10.1001/jamanetworkopen.2021.5832

ATTACHMENT A:

PATHWAY FOR SUSPECTED PEDIATRIC MALTREATMENT

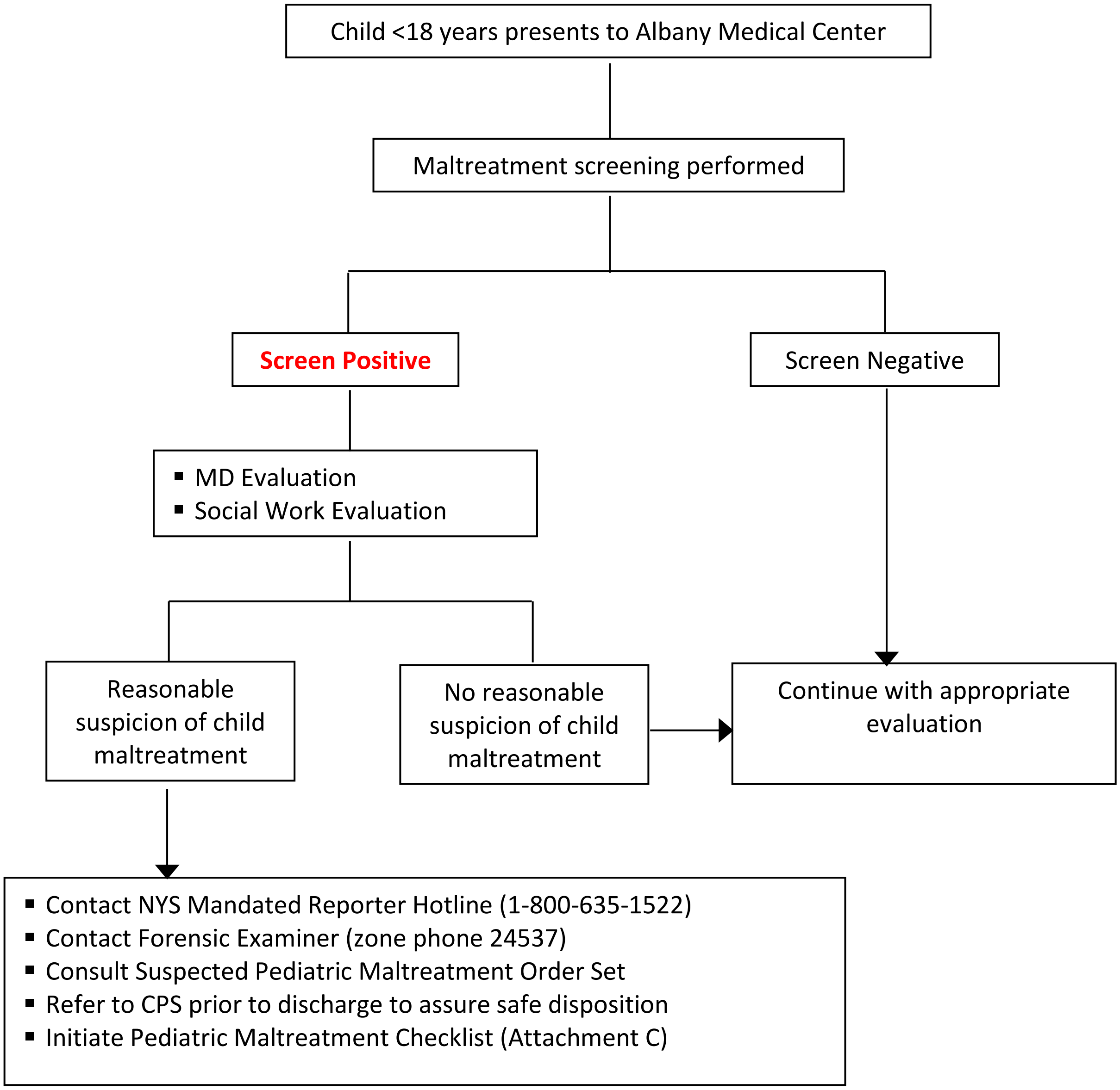

09/2018; 11/2019; 1/2020, 4/2020, 6/2021, 10/2022

Attachment B
Suspected Pediatric Maltreatment Order Set

Consideration for Drop Off Criteria

1. *Distal Radial or Ulnar Buckle Fracture in a Cruising Child >9 Month with a History of a Consistent Fall*
2. *Linear, Unilateral, Non-Depressed Skull Fracture in a Child > 6 Month with a History of a Significant Fall and No Additional Clinical Concerns.*
3. *Unilateral Clavicle Fracture Likely Attributed to Birth (Acute Fracture in Infants < 22 Days or Healing Fracture in Infants < 30 Days)*

Pediatric Maltreatment Protocol

All Patients

- ☑ Vital Signs
- ☑ Obtain Height
- ☑ Obtain Weight
- ☐ Head Circumference *If <24 Months*
- ☑ Social Work Consult, Other Specify, Maltreatment Screening
- ☑ Nurse Forensic Examination
- ☐ Notify NYS Mandated Reporter Hotline (1-800-635-1522)

All Admitted Patients

- ☑ Physician Consult, Trauma

☐ Patients < 6 Months

May Wait Until the Next day if Child Admitted

- ☑ Skeletal Survey Infant Less than 30 Days, Peds
- ☑ Skeletal Survey, Complete, Peds
- ☑ CT Head, Peds
- ☑ CT 3D Rendering, Peds

If Serious Head Injury and High Index of Suspicion for Abusive Head Trauma

- ☐ MRI Brain, Peds
- ☐ MRI Spine Cervical, Peds
- ☐ MRI Spine Lumbar, Peds
- ☐ MRI Spine Thoracic, Peds

If AST or ALT >=80

- ☑ CT Abdomen with Contrast, Peds

Ophthalmological Exam: Only with the presence of an Intracranial Hemorrhage or skull fracture on CT scan, Neurosurgery must clear patient for eye exam.

Ophthalmology Consult may wait until next day if clinically asymptomatic. Must call Ophthalmology for consult.

- ☑ Physician Consult, Ophthalmology
- ☑ Comprehensive Metabolic Panel
- ☑ CBC without Differential
- ☑ Lipase

- ☐ **Patients 6-12 Months**

  May Wait Until the Next Day if Child Admitted
  - ☑ Skeletal Survey, Complete, Peds

  If Facial Bruising, Abnormal Neurological Exam, Any Head or Facial Injury or Clinical Concern
  - ☑ CT Head, Peds
  - ☑ CT 3D Rendering, Peds

  If Serious Head Injury and High Index of Suspicion for Abusive Head Trauma
  - ☐ MRI Brain, Peds
  - ☐ MRI Spine Cervical, Peds
  - ☐ MRI Spine Lumbar, Peds
  - ☐ MRI Spine Thoracic*, Peds*

  If AST or ALT >=80
  - ☑ *CT Abdomen with Contrast, Peds*

  Ophthalmological Exam: Only with the presence of an Intracranial Hemorrhage or Skull Fracture on CT, Neurosurgery must clear patient for eye exam.

  Ophthalmology Consult may wait until next day if clinically asymptomatic. Must call Ophthalmology for consult.
  - ☑ Physician Consult, Ophthalmology
  - ☑ Comprehensive Metabolic Panel
  - ☑ CBC without Differential
  - ☑ Lipase

- ☐ **Patients 12-24 Months**

  May Wait Until the Next Day if Child Admitted
  - ☑ Skeletal Survey, Complete, Peds

  If Facial Bruising, Abnormal Neurological Exam, Any Head or Facial Injury or Clinical Concern
  - ☑ CT Head, Peds
  - ☑ CT 3D Rendering, Peds

  If Serious Head Injury and High Index of Suspicion for Abusive Head Trauma
  - ☐ MRI Brain, Peds
  - ☐ MRI Spine Cervical, Peds
  - ☐ MRI Spine Lumbar, Peds
  - ☐ MRI Spine Thoracic, Peds

  If AST or ALT >=80
  - ☑ *CT Abdomen with Contrast, Peds*

  Ophthalmological Exam: Only with the presence of an Intracranial Hemorrhage or Skull Fracture on CT. Neurosurgery must clear patient for eye exam.

  Ophthalmology Consult may wait until next day if clinically asymptomatic. Must call Ophthalmology for consult.
  - ☑ Physician Consult, Ophthalmology
  - ☑ Comprehensive Metabolic Panel
  - ☑ CBC without Differential
  - ☐ Lipase

☐ **Patients 24-36 Months**

Strongly Consider if Seriously Injured, May Wait Until the Next Day if Child Admitted

☑ Skeletal Survey, Complete, Peds

If Abnormal Neurological Exam, or Other Clinical Concern

☑ CT Head, Peds

☑ CT 3D Rendering, Peds

If AST or ALT >=80

☑ CT Abdomen with Contrast, Peds

Strongly Consider

☑ Comprehensive Metabolic Panel

If Bruising or Intracranial Hemorrhage

☑ CBC without Differential

Strongly Consider

☑ Lipase

☐ **Patients > 36 Months**

Orders based on Provider Discretion and Clinical Indications

Portable in Pediatric Zone

☐ Chest, Portable, Peds, Emergent

☐ Pelvis, 1 to 2 Views, Portable, Peds, Emergent

☐ Spine, Cervical Portable, Peds, Emergent

Room A1 and A2 ONLY

☐ Chest, 1 View, Peds, Emergent

☐ Chest, PA and Lateral, Peds, Emergent

☐ Abdomen, 2 Position, Peds, Emergent

☐ Pelvis, 1 to 2 Views, Peds, Emergent

☐ Spine, Cervical 2 to 3 Views, Routine, Peds, Emergent

CT Exams

☐ CT Head, Peds, Emergent

☐ CT Orbits, Peds, Emergent

☐ CT Temporal Bones, Peds, Emergent

☐ CT MaxilloFacial, Face, Peds, Emergent

☐ CT Spine Cervical, Peds, Emergent

☐ CT Chest, Peds, Emergent

☐ CT Chest, Abdomen, Pelvis with Contrast, Peds, Emergent

☐ CT Abdomen, Peds, Emergent

☐ CT Abdomen and Pelvis without Contrast, Peds, Emergent

☐ CT Pelvis, Peds, Emergent

Internal use only, NOT part of the chart. When patient is discharged please give to social worker for review

Patient Label

Attachment C

Pediatric Maltreatment Checklist

The objective of the checklist is to streamline communication for all providers covering the NAT patient. Refer to the ***Pediatric Maltreatment Protocol*** for policies, procedures and order set.

**Has social work been contacted?**	Name of ED social worker: ________________<br>Name of inpatient social worker: ________________<br>Phone Number: ________________
**Has the forensic examiner been contacted?**<br>**What date/time was the exam performed?**	Name of forensic examiner:<br>Date of exam: __________ Time of exam: ________
**Has Child Protective Services been called?**	Date/Time called: ________________<br>Name of CPS caseworker: ________________<br>Phone number: ________________<br>County involved: ________________
**Has law enforcement been notified?**	Date/Time called: ________________<br>Law Enforcement Agency: ________________<br>Detective assigned: ________________<br>Phone#: ________________
**Admitting Service:**	Attending Physician: ________________
**Test Completed**	1.________________<br>2.________________<br>3.________________<br>4.________________<br>5.________________
**Injuries Identified**	1.________________<br>2.________________<br>3.________________<br>4.________________<br>5.________________
**Consulting services**	1.____________Date/Time____________<br>2.____________Date/Time____________<br>3.____________Date/Time____________<br>4.____________Date/Time____________

Discharge Criteria

- **Is the patient's discharge order placed?**
- **Are all final interpretations of all imaging/consultations in the medical record, including Ophthalmology?**
- **Are the injuries concerning for maltreatment?**
- **Has it been relayed to CPS injuries are concerning for maltreatment?**
